# Supplementary figures and images for: Bone Status in Patients with Phenylketonuria: A Systematic Review
Source: Nutrients. 2020 Jul 20;12(7):2154. doi: 10.3390/nu12072154 (PMC7400926; doi:10.3390/nu12072154)

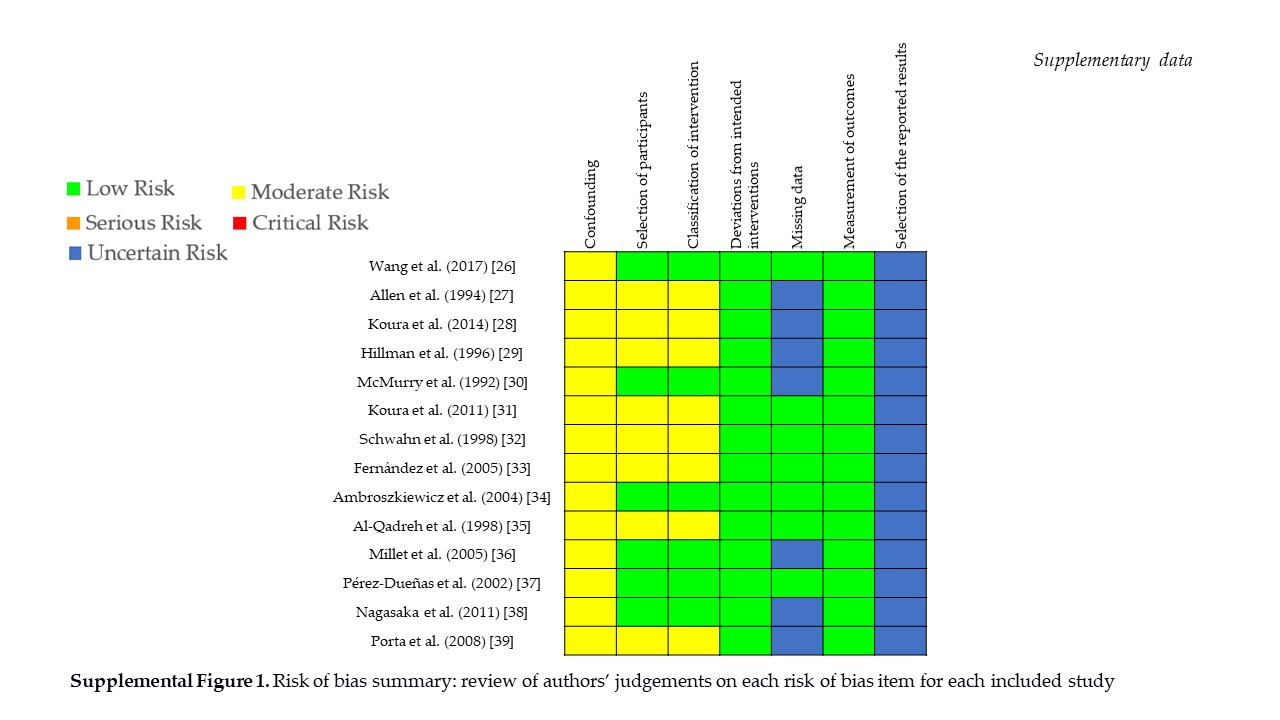

Supplement: Supplementary file 1 [file nutrients-12-02154-s001.zip › Supplemental figure 1.jpg]

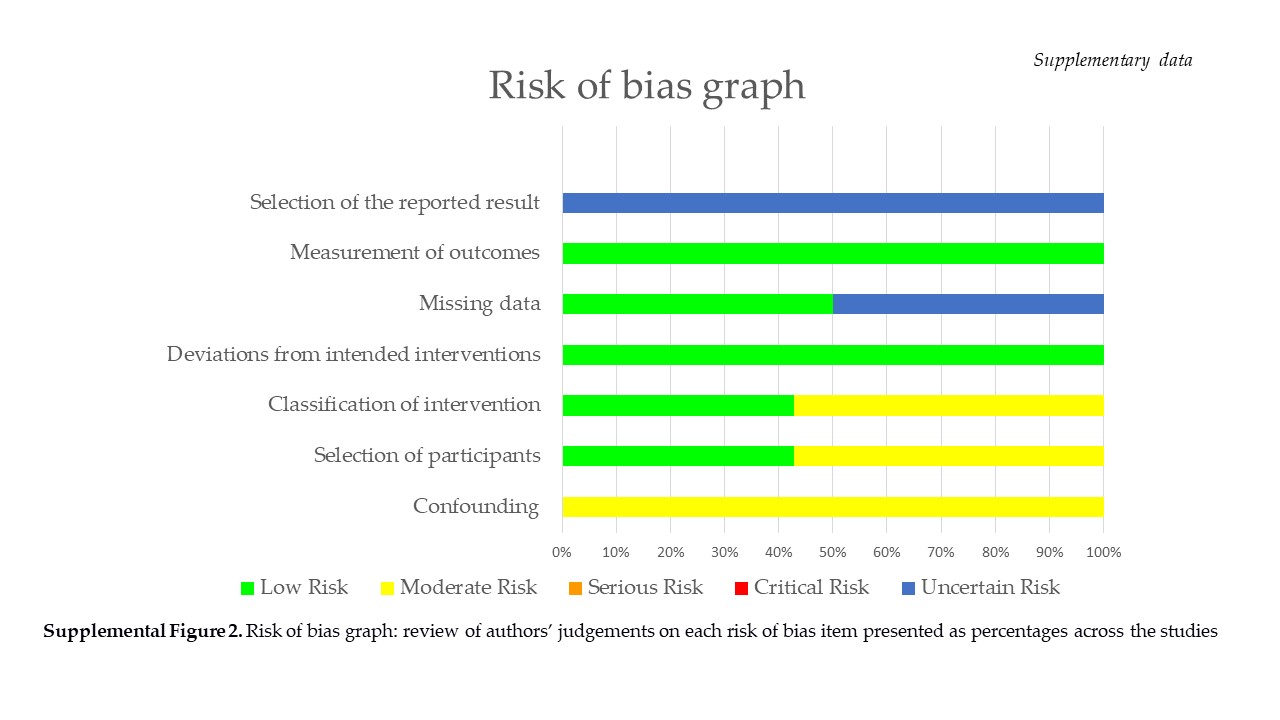

Supplement: Supplementary file 1 [file nutrients-12-02154-s001.zip › Supplemental figure 2.jpg]
